# Superbubbles revisited

**DOI:** 10.1186/s13015-018-0134-3

**Published:** 2018-12-01

**Authors:** Fabian Gärtner, Lydia Müller, Peter F. Stadler

**Affiliations:** 1 Competence Center for Scalable Data Services and Solutions Dresden/Leipzig, Universität Leipzig, Augustusplatz 12, 04107 Leipzig, Germany; 2 Bioinformatics Group, Department of Computer Science, Universität Leipzig, Härtelstraße 16–18, 04107 Leipzig, Germany; 3 Interdisciplinary Center for Bioinformatics, Universität Leipzig, Härtelstraße 16–18, 04107 Leipzig, Germany; 4 Natural Language Processing Group, Department of Computer Science, Universität Leipzig, Augustusplatz 12, 04107 Leipzig, Germany; 5 Max Planck Institute for Mathematics in the Sciences, Inselstraße 22, 04103 Leipzig, Germany; 6 Fraunhofer Institute for Cell Therapy and Immunology, Perlickstraße 1, 04103 Leipzig, Germany; 7 Department of Theoretical Chemistry, University of Vienna, Währinger Straße 17, 1090 Vienna, Austria; 8 Center for Non-coding RNA in Technology and Health, Grønegårdsvej 3, 1870 Frederiksberg C, Denmark; 9 Santa Fe Institute, 1399 Hyde Park Rd., Santa Fe, NM 87501 USA

**Keywords:** Superbubble, de Bruijn graph, Genome assembly, Linear time algorithm

## Abstract

**Background:**

Superbubbles are distinctive subgraphs in direct graphs that play an important role in assembly algorithms for high-throughput sequencing (HTS) data. Their practical importance derives from the fact they are connected to their host graph by a single entrance and a single exit vertex, thus allowing them to be handled independently. Efficient algorithms for the enumeration of superbubbles are therefore of important for the processing of HTS data. Superbubbles can be identified within the strongly connected components of the input digraph after transforming them into directed acyclic graphs. The algorithm by Sung et al. (IEEE ACM Trans Comput Biol Bioinform 12:770–777, 2015) achieves this task in $\mathcal {O}(m~log(m))$-time. The extraction of superbubbles from the transformed components was later improved to by Brankovic et al. (Theor Comput Sci 609:374–383, 2016) resulting in an overall $\mathcal {O}(m+n)$-time algorithm.

**Results:**

A re-analysis of the mathematical structure of superbubbles showed that the construction of auxiliary DAGs from the strongly connected components in the work of Sung et al. missed some details that can lead to the reporting of false positive superbubbles. We propose an alternative, even simpler auxiliary graph that solved the problem and retains the linear running time for general digraph. Furthermore, we describe a simpler, space-efficient $\mathcal {O}(m+n)$-time algorithm for detecting superbubbles in DAGs that uses only simple data structures.

**Implementation:**

We present a reference implementation of the algorithm that accepts many commonly used formats for the input graph and provides convenient access to the improved algorithm. https://github.com/Fabianexe/Superbubble.

## Background

Under idealizing assumption, the genome assembly problem reduces to finding an Eulerian path in the de Bruijn graph [1] that represents the collection of sequencing reads [2]. In real-life data sets, however, sequencing errors and repetitive sequence elements contaminate the de Bruijn graph with additional, false, vertices and edges. Assembly tools therefore employ filtering steps that are based on recognizing local motifs in the de Bruijn graphs that correspond to this kind of noise, see e.g. [3]. Superbubbles also appear naturally in the multigraphs in the context of supergenome coordinatization [4], i.e., the problem of finding good common coordinate systems for multiple genomes.

The simplest such motif is a *bubble*, comprising two or more isolated paths connecting a source *s* to a target *t*, see [5] for a formal analysis. While bubbles are easily recognized, most other motives are much more difficult to find. Superbubbles are a complex generalization of bubbles that were proposed in [6] as an important class of subgraphs in the context of HTS assembly. It will be convenient for the presentation in this paper to first consider a more general class of structure which are obtained by omitting the minimality criterion:

### **Definition 1**

(*Superbubbloid*) Let $G = (V,E)$ be a directed multi-graph and let (*s*, *t*) be an ordered pair of distinct vertices. Denote by $U_{st}$ the set of vertices reachable from *s* without passing through *t* and write $U^+_{ts}$ for the set of vertices from which *t* is reachable without passing through *s*. Then the subgraph $G[U_{st}]$ induced by $U_{st}$ is a *superbubbloid* in *G* if the following three conditions are satisfied:

1. $t\in U_{st}$, i.e., *t* is reachable from *s* (reachability condition).
2. $$U_{st}=U^+_{ts}$$ (matching condition).
3. $G[U_{st}]$ is acyclic (acyclicity condition).

We call *s*, *t*, and $U_{st}\backslash\{s,t\}$ the *entrance*, *exit*, and *interior* of the superbubbloid. We denote the induced subgraph $G[U_{st}]$ by $\langle s,t\rangle$ if it is a superbubbloid with entrance *s* and exit *t*.

A superbubble is a superbubbloid that is minimal in the following sense:

### **Definition 2**

A superbubbloid $\langle s,t\rangle$ is a superbubble if there is no $s'\in U_{st}\backslash\{s\}$ such that $\langle s',t\rangle$ is a superbubbloid.

We note that Definition 2 is a simple rephrasing of the language used in [6], where a simple $\mathcal {O}(n(m+n))$-time algorithm was proposed that, for each candidate entrance *s*, explicitly retrieves all superbubbles $\langle s,t\rangle$. Since the definition is entirely based on reachability, multiple edges are irrelevant and can be omitted altogether. Hence we only consider simple digraphs throughout.

The vertex set of every digraph *G*(*V*, *E*) can be partitioned into its strongly connected components. Denote by ${\bar{V}}$ the set of singletons, i.e., the strongly connected components without edges. One easily checks that the induced subgraph $G[{\bar{V}}]$ is acyclic. Furthermore, denote by $\mathfrak {S}$ the partition of *V* comprising the non-singleton connected components of *G* and the union ${\bar{V}}$ of the singleton. The key observation of [7] can stated as

### **Proposition 1**

*Every superbubble* $\langle s,t\rangle$* in G (V , E ) is an induced subgraph of G [C] for some*$C\in \mathfrak {S}$.

It ensures that it is sufficient to search separately for superbubbles within *G*[*C*] for $C\in \mathfrak {S}$. However, these induced subgraphs may contain additional superbubbles that are created by omitting the edges between different components. In order to preserve this information the individual components *C* are augmented by artificial vertices [7]. The augmented component *C* is then converted into a directed acyclic graph (DAG). Within each DAG the superbubbles can be enumerated efficiently. With the approach of [7], this yields an overall $\mathcal {O}(m\log m)$-time algorithm, the complexity of which is determined by the extraction of the superbubbles from the component DAGs. The partitioning of *G*(*V*, *E*) into the components *G*[*C*] for $C\in \mathfrak {S}$ and the transformation into DAGs can be achieved in $\mathcal {O}(m+n)$-time. Recently, Brankovic et al. [8] showed that superbubbles can be found in linear time within a DAG. Their improvement uses the fact that the DAG can always be topological ordered in such a way that superbubbles appears as a contiguous blocks. In this ordering, furthermore, the candidates for entrance and exit vertices can be narrowed down considerably. For each pair of entrance and exit candidates (*s*, *t*), it can then be decided in constant time whether $U_{st}$ is indeed a superbubble. Using additional properties of superbubbles to further prune the candidate list of (*s*, *t*) pairs results in $\mathcal {O}(m+n)$-time complexity.

The combination of the work of [7] with the improvement of [8] results in the state of the art algorithm. The concept of a superbubble was extended to bi-directed and bi-edged graphs, called ultrabubble in [9–11]. The enumeration algorithm for ultrabubbles in [9] has a worst case complexity of $\mathcal {O}(mn)$, and hence does not provide an alternative for directed graphs.

**Fig. 1:**
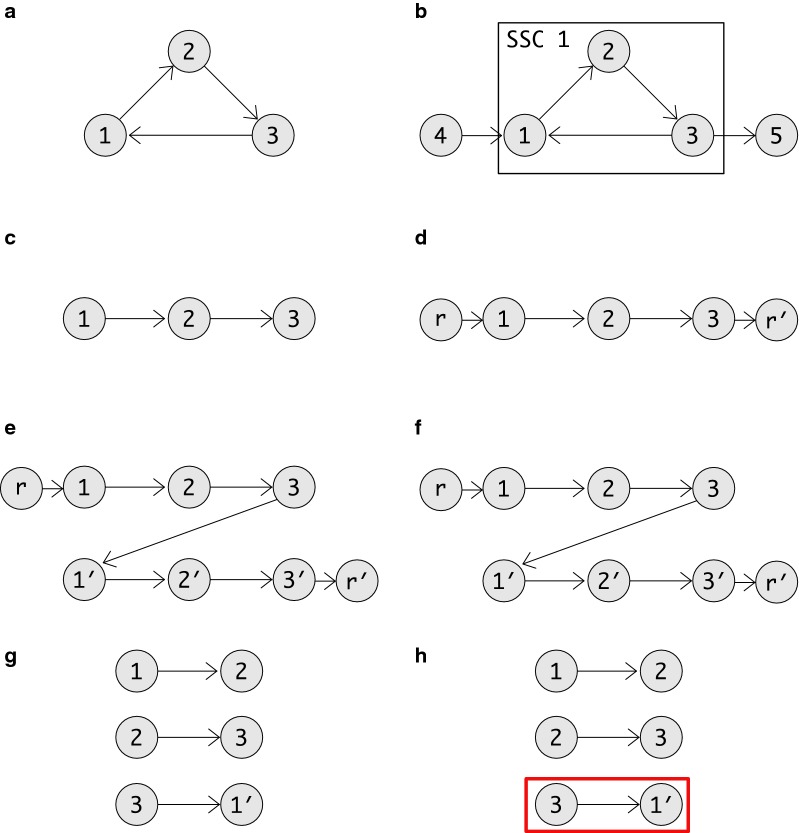
False-positive “superbubble” returned by the algorithm of Sung et al. [7]. The directed 3-cycle **a** on the l.h.s. correctly yields the three subgraphs on two vertices as superbubbles. The graph **b** on the r.h.s., on the other hand, includes **a** as the only non-trivial strongly connected component. The vertices 1 and 3 have additional neighbors which are replaced by artificial nodes *r* and $r'$, respectively. **c**, **d** are the corresponding DFS trees using an artificial source as root. Since no artificial source is present in **a**, a random vertex, here 1, is used as root. The correspond DAGs in **e**, **f** are constructed from duplicate copies of the DFS trees, augmented by source and sink vertices in **e** since these were lacking in **c**. Note that the same DAGs (**g, h**) are obtained for **a** and the non-trivial copy of **a** in **b**. Hence the same superbubbles are returned in both cases. While $\langle 3,1\rangle$ is a valid result for **a**, it is a false positive for **b** since 3 is not a valid entrance and 1 is not a valid exit in **b**

A careful analysis showed that sometimes false-positive superbubbles are reported, see Fig. 1. These do not constitute a fatal problem because they can be recognized easily in linear total time simply by checking the tail of incoming and head of outgoing edges. It is nevertheless worth while to analyse the issue and to seek a direct remedy. As we shall see below, the false positive subgraphs are a consequence of the way in which a strongly connected component *C* is transformed into a two DAGs that are augmented by either the source or target vertices.

## Theory

In the first part of this section we revisit the theory of superbubbles in digraphs in some more detail. Although some of the statements below have appeared at least in similar for in the literature [6–8] we give concise proofs and take care to disentangle properties that depend on minimality from those that hold more generally. This refined mathematical analysis sets the stage in the second part for identifying the reason for the problems with the auxiliary graph constructed in [7] shows how the problem can be solved efficiently in these cases using an even simpler auxiliary graph. In the third part we elaborate on the linear time algorithm on [8] for DAGs. We derive a variant that has the same asymptotic running time but seems easier to explain.

### Weak superbubbloids

Although we do not intend to compute superbubbloids in practice, they feature several convenient mathematical properties that will simplify the analysis of superbubbles considerably. The main aim of this section is to prove moderate generalizations of the main results of [6, 7]. To this end, it will be convenient to rephrase the reachability and matching conditions (S1) and (S2) for the vertex set *U* of superbubbloid with entrance *s* and exit *t* in the following, a more expanded form.

#### **Lemma 1**

*Let G be a digraph,* $U\subset V(G)$ *and* $s,t\in U.$ *Then (S1) and (S2) holds for* $U=U_{st}=U^+_{ts}$ *if and only if the following four conditions are satisfied*

(S.i): *Every* $u\in U$ *is reachable from s.*
(S.ii): *t is reachable from every* $u\in U$.
(S.iii): *If *$u\in U$* and *$w\notin U$* then every* $w\rightarrow u$ *path contains s*.
(S.iv): $u\in U$ *and* $w\notin U$ *then every* $u\rightarrow w$ *path contains t.*

#### *Proof*

Suppose (S1) and (S2) are true. Then $u\in U_{st}$ and $u\in U^+_{ts}$ implies, by definition, that *u* is reachable from *s*, i.e. (S.i) and (S.ii) holds. By (S2) we have $U:=U_{st}=U^+_{ts}$. If $w\notin U$ it is not reachable from *s* without passing through *t*. Since every *u* is reachable from *s* without passing through *t*, we would have $w\in U$ if *w* was reachable from any $u\in U$ on a path not containing *t*, hence (S.iv) holds. Similarly, since *t* is reachable from *u* without passing through *s*, we would have $w\in U$ if *v* could be reached from *w* along a path that does not contain *s*, i.e. (S.iii) holds.

Now suppose (S.i), (S.ii), (S.iii), and (S.iv) holds. Clear, both (S.i) and (S.ii) already imply (S1). Since $u\in U$ is reachable from *s* by (S.ii) and every path reaching $w\notin U$ pases through *t* by (S.iii), we have $U=U_{st}$. By (S.i), *t* is reachable from every $u\in U$ and by (S.iv) *t* can by reached from $w\notin U$ only by passing through *s*, i.e., $U=U^+_{ts}$, i.e., $U_{st}=U^+_{ts}$. $\square$

#### **Corollary 1**

*Suppose U, s, and t satisfies (S.i), (S.ii), (S.iii), and (S.iv). Then every path connecting s to* $u\in U$ *and u to t is contained within U.*

#### *Proof*

Assume, for contradiction, that there $u\rightarrow t$ path containing a vertex $w\notin \langle s,t\rangle.$ By definition of the set $U_{st},$ $w\notin U_{st}$ is not reachable from $u\in U_{st}$ without passing through *t* first, i.e., *w* cannot be part of a $u\rightarrow t$ path. $\square$

Corollary 1 shows that subgraphs satisfying (S1) and (S2) related to reachability structures explored in some detail in [12, 13]. In the following it will be useful to consider

(S.v): If (*u*, *v*) is an edge in *U* then every $v\rightarrow u$ path in *G* contains both *t* and *s*.

In the following we shall see that (S.v) acts a slight relaxation of the acyclicity axiom ((S3)).

#### **Lemma 2**

*Let G(V, E) be a digraph,* $U\subseteq V$ *and* $s,t\in U.$

*If U is the vertex set of the superbubbloid* $\langle s,t\rangle,$ *it satisfies (S.v).*

*If U satisfies (S.i), (S.ii), (S.iii), (S.iv), and (S.v), then* $G[U]\backslash\{(t,s)\},$ *the subgraph induced by U without the potential edge from t to s, is acyclic.*

#### *Proof*

Suppose *U* is the vertex set of a superbubbloid with entrance *s* and exit *t*. If (*u*, *v*) is an edge in *U*, then $v\ne s$ by (S3). Since *v* is reachable from *s* within *U*, no $v\rightarrow s$ path can exist within *U*, since otherwise there would be a cycle, contradicting (S3), that any $v\rightarrow s$ path passes through *t*. There are two cases: If there $(t,s)\in E$, any path containing this edge trivially contains both *s* and *t*. The existence of the edge (*t*, *s*) contradicts (S3). Otherwise, any $t\rightarrow s$ path contain at least one vertex $x\notin U$. By (S.iii) and (S.iv) every $v\rightarrow x$ path contains *t* and every $x\rightarrow u$ path contain *s* and *t*, respectively. Hence the first statement holds.

Conversely, suppose (S.v) holds, i.e., every directed cycle *Z* within *U* contains *s* and *t*. Suppose (*t*, *s*) is not contained *Z*, i.e., there is vertex $u\in U\backslash\{s,t\}$ such that $(t,u)\in E$. By (S.ii), *t* is reachable from *u* without passing through *s*, and every $u\rightarrow t$ path is contained in *U* by Corollary 1. Thus there is a directed cycle within *U* that contains *u* and *t* but not *s*, contradicting (S.v). Removing the edge (*t*, *s*) thus cuts every directed cycle within *U*, and hence $G[U]\backslash\{(t,s)\}$ is acyclic. $\square$

Although the definition of [6] (our Definition 2) is also used in [7], the notion of a superbubble is tacitly relaxed in [7] by allowing an edge (*t*, *s*) from exit to entrance, although this contradicts the acyclicity condition (S3). This suggests

#### **Definition 3**

(*Weak Superbubbloid*) Let *G*(*V*, *E*) be a digraph, $U\subseteq V$ and $s,t\in U$. The subgraph *G*[*U*] induced by *U* is a *weak superbubbloid* if U satisfies (S.i), (S.ii), (S.iii), (S.iv), and (S.v).

We denote a weak superbubbloids with entrance *s* and exit *t* by $\langle s,t\rangle$ and write $U_{st}$ for its vertex set. As an immediate consequence of Definition 3 and Lemma 2 we have

#### **Corollary 2**

*A weak superbubbloid *$\langle s,t\rangle$ *is a superbubbloid in G(V, E) if and only if *$(t,s)\notin E$.

The possibility of an edge connecting *t* to *s* will play a role below, hence we will focus on weak superbubbloids in this contribution.

First we observe that a weak superbubbloids contained within another weak superbubbloid must be a superbubbloid because the existence of an edge from exit to entrance contradicts (S.v) for the surrounding weak superbubbloid. We record this fact as

#### **Lemma 3**

*If* $\langle s,t\rangle$ *and* $\langle s',t'\rangle$ *are weak superbubbloids with* $s',t'\in \langle s,t\rangle$ *and* $\{s',t'\}\ne \{s,t\},$ *then* $\langle s',t'\rangle$ *is a superbubbloid.*

The result will be important in the context of minimal (weak) superbubbloids below.

Another immediate consequence of Lemma 2 is

#### **Corollary 3**

*Let* $\langle s,t\rangle$ *be a weak superbubbloid in G. If there is an edge (u, v) in* $\langle s,t\rangle$ *that is contained in a cycle, then every edges in* $\langle s,t\rangle$ *is contained in cycle containing s and t.*

#### *Proof*

By (S.v) there is cycle running though *s* and *t*. Let (*u*, *v*) be an edge in $\langle s,t\rangle$. Since *u* is reachable from *s* and *v* reaches *t* within *U*, there is a cycle containing *s*, *t*, and the edge (*u*, *v*). $\square$

#### **Theorem 1**

*Every weak superbubbloid* $\langle s,t\rangle$ *in G(V, E) is an induced subgraph of G[C] for some* $C\in \mathfrak {S}.$

#### *Proof*

First assume that $\langle s,t\rangle$ contains an edge (*u*, *v*) that is contained in cycle. Then by (S.v), there is cycle through *s* and *t* and thus in particular a (*t*, *s*) path. For every $u\in U$, there is a path within *U* from *s* to *t* through *u* by (S.i), (S.ii), and Lemma 1. Thus $\langle s,t\rangle$ is contained as an induced subgraph in a strongly connected component *G*[*C*] of *G*. If there is no edge in $\langle s,t\rangle$ that is contained in a cycle, then every vertex in $\langle s,t\rangle$ is a strongly connected component on its own. $\langle s,t\rangle$ is therefore an induced subgraph of $G[{\bar{V}}]$. $\square$

Theorem 1 establishes Proposition 1, the key result of [7], in sufficient generality for our purposes. Next we derive a few technical results that set the stage for considering minimality among weak superbubbloids.

#### **Lemma 4**

*Assume that* $\langle s,t\rangle$ *is a weak superbubbloid and let u be an interior vertex of* $\langle s,t\rangle.$ *Then* $\langle s,u\rangle$ *is a superbubbloid if and only if* $\langle u,t\rangle$ *is a superbubbloid.*

#### *Proof*

Suppose $\langle s,u\rangle$ is a superbubbloid. Set $W_{ut}:=(U_{st}\backslash U_{su})\cup \{u\}$ and consider $w\in W_{ut}.$ The subgraph induced by $W_{ut}$ is an induced subgraph of $\langle s,t\rangle \backslash\{(t,s)\}.$ Hence it is acyclic and in particular $(t,u)\notin E,$ i.e., (S.v) and even (S3) holds. Since $t\notin U_{su}$ every path from *s* to *t* runs through *u*. Since *w* is reachable from *s* there is a path from *s* through *u* to *w*, i.e., *w* is reachable from *u*. Thus (S.i) holds. (S.ii) holds by assumption since *t* is reachable from *w*. Now suppose $v\notin W_{ut}$ and $w\in W_{ut}.$ If $v\notin U_{st},$ then every $v\rightarrow w$ path passes through *s* and then through *u*, the exit of $\langle s,u\rangle$ before reaching *w*. If $v\in U_{st},$ then $v\in U_{su}\backslash\{u\}$ and thus every $v\rightarrow w$ path passes through *u* as the exit of $\langle s,u\rangle.$ Hence $W_{ut}$ satisfied (S.iii). If $v\in U_{st},$ then $v\in U_{su}\backslash\{u\}$ and thus every $w\rightarrow v$ path passes through *s*. By (S.v) there is no $w\rightarrow s$ path within $\langle s,t\rangle \backslash\{(t,s)\},$ and thus any $w\rightarrow v$ includes (*t*, *s*) or a vertex $y\notin U_{st}.$ By construction, all $w\rightarrow y$ paths contain *t*, and thus all $w\rightarrow v$ paths also pass through *t* and $W_{ut}$ also satisfies (S.iv).

Conversely suppose $\langle u,t\rangle$ is a superbubbloid. We have to show that $W_{su}:=(U_{st}\backslash U_{ut})\cup \{u\}$ induces a superbubbloid. The proof strategy is very similar. As above we observe that (S.v), (S.i), and (S.ii) are satisfied. Now consider $v\notin W_{su}$ and $w\in W_{su}.$ If $v\notin U_{st}$ then every $v\rightarrow w$ path contains *s*; otherwise $v\in U_{ut}\backslash\{u\}$ and $v\rightarrow w$ passes through *t* and thus also through *s* by Corollary 1, thus (S.iii) holds. If $v\in U_{st},$ then $v\in U_{ut}\backslash\{u\},$ in which case every $w\rightarrow v$ path passes through *u*. Otherwise $v\notin U_{st}$ then every $w\rightarrow v$ runs through $t\in U_{st}$ and thus in particular also through *u*. Hence (S.iv) holds. $\square$

#### **Lemma 5**

*Let* $\langle w,u\rangle$ *and* $\langle s,t\rangle$ *be two weak superbubbloids such that u is an interior vertex of* $\langle s,t\rangle,$ *s is an interior vertex of* $\langle w,u\rangle,$ *w is not contained in* $\langle s,t\rangle$ *and t is not contained in* $\langle w,u\rangle.$ *Then the intersection* $\langle s,u\rangle =\langle w,u\rangle \cap \langle s,t\rangle$ *is also a superbubbloid.*

#### *Proof*

First consider the intersection $\langle s,u\rangle.$ $u\in \langle s,t\rangle$ is reachable from *s*, hence (S1) holds. Furthermore $\langle s,u\rangle$ is an induced subgraph of $\langle s,t\rangle \backslash\{(t,s)\}$ and hence again acyclic (S3). Set $W_{su}:=U_{wu}\cap U_{st}$ and consider $v\in W_{su}.$ First we note that *v* is reachable from *s* by definition of $\langle s,t\rangle$ and *u* is reachable from *v* by definition of $\langle w,u\rangle.$ Let $x\notin W_{su}$ and $v\in W_{su}.$ If $x\notin U_{st}$ then every $x\rightarrow v$ path passes through *s*; if $x\in U_{st}$ then $x\notin U_{wu}$ (and $v\in U_{wu}$) and thus every $x\rightarrow v$ path passes through *w*. Since $w \notin U_{st},$ we know that every $x\rightarrow v$ path contains *s*.

If $x\notin U_{wu},$ then every $v\rightarrow x$ path passes through *u*; otherwise $x\in U_{wu}$ but $x\notin U_{st},$ thus every $v\rightarrow x$ path passes through $t\notin U_{wu}$ and hence also through *u*. Thus $W_{su}$ is a superbubbloid. $\square$

We include the following result for completeness, although it is irrelevant for the algorithmic considerations below.

#### **Lemma 6**

*Let* $\langle w,u\rangle$ *and* $\langle s,t\rangle$ *be defined as in Lemma* 5 *. Then the union* $\langle w,t\rangle =\langle w,u\rangle \cup \langle s,t\rangle$ *is superbubbloid if and only if the induced subgraph* $\langle w,t\rangle$ *satisfies (S.v).*

#### *Proof*

Since $\langle w,s\rangle,$ $\langle s,u\rangle,$ $\langle u,t\rangle$ are superbubbloids, *t* is reachable from *w*, i.e., (S1) holds. By the same token, every $v\in W_{wt}:=U_{wu}\cup U_{st}$ is reachable from *w* or *s* and reaches *u* or *t*. Since *s* is reachable from *w* and *t* is reachable from *u*, every $v\in W_{wt}$ is reachable from *w* and reaches *t*. Now consider $x\notin W_{wt}$ and $v\in W_{wt}$. If $v\in U_{wu}$ every $x\rightarrow v$ path passed through *w*; if $v\in U_{s,t}$, it passes through $s\in U_{wu}$ and thus also through *w*. If $v\in U_{st}$, then every $v\rightarrow x$ path passed through *t*. If $v\in U_{wu}$ it passes through $u\in U_{st}$ and thus also through *t*. Thus $W_{wt}$ satisfies (S2). Thus $\langle w,t\rangle$ is a weak superbubbloid if and only if (S.v) holds. $\square$

#### **Lemma 7**

*Let* $\langle s,t\rangle$ *be a weak superbubbloid in G with vertex set* $U_{st}.$ *Then* $\langle s,t\rangle$ *is a weak superbubbloid in the induced subgraph G[W] whenever* $U_{st}\subseteq W.$

#### *Proof*

Conditions (S.i), (S.ii), and (S.v) are trivially conserved when *G* is restricted to *G*[*W*]. Since every $w\rightarrow u$ and $u\rightarrow w$ path with $u\in U_{st}$ and $w\notin U_{st}$ within *W* is also such a path in *V*, we conclude that (S.iii) and (S.iv) are satisfied w.r.t. *W* whenever they are true w.r.t. the larger set *V*. $\square$

The converse is not true. The restriction to induced subgraphs thus can introduce additional (weak) superbubbloids. As the examples in Fig. 1 show, it is also possible to generate additional superbubbles.

Finally we turn our attention to the minimality condition.

#### **Definition 4**

A weak superbubbloid $\langle s,t\rangle$ is a *weak superbubble* if there is no interior vertex $t'$ in $\langle s,t\rangle$ such that $\langle s,t'\rangle$ is a weak superbubbloid.

The “non-symmetric” phrasing of the minimality condition in Definitions 2 and 4 [6–8] is justified by Lemma 4: If $\langle s,t\rangle$ and $\langle s,t'\rangle$ with $t'\in \langle s,t\rangle$ are superbubbloids, then $\langle t',t\rangle$ is also a superbubbloid, and thus $\langle s,t\rangle$ is not a superbubble. As a direct consequence of Lemma 3, furthermore, we have

#### **Corollary 4**

*Every superbubble is also a weak superbubble.*

Lemma 4 also implies that every weak superbubbloid, which is not a superbubble itself, can be decomposed into consecutive superbubbles:

#### **Corollary 5**

*If* $\langle s,t\rangle$ *is a weak superbubbloid, then it is either a weak superbubble or there is a sequence of vertices* $v_k$ *with* $s=v_1,v_2,\dots ,v_k=t,$ $k\ge 3,$ *such that* $\langle v_i,v_{i+1}\rangle$ *is a superbubble for all* $i\in \{1,2,\dots , k-1\}.$

**Fig. 2:**
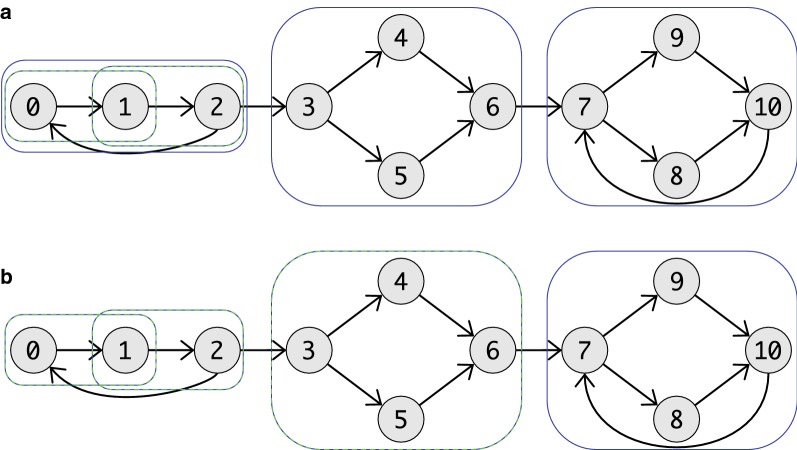
An example graph with in which all (weak) superbubbloids and (weak) superbubbles are marked. In **a** are all weak superbubbloids (blue) and all superbubbloids (green) marked. Note that beside $\langle 0,2\rangle$ and $\langle 7,10\rangle$ all weak superbubbloids are also superbubbloids. In **b** are all weak superbubbles (blue) and all superbubbles (green) marked. The weak superbubbloids $\langle 0,2\rangle$ is the only superbubbloids that creates no (weak) superbubble. So that $\langle 7,10\rangle$ is the only superbubble that is not a weak superbubble

A useful consequence of Lemma 5, furthermore, is that superbubbles cannot overlap at interior vertices since their intersection is again a superbubbloid and thus neither of them could have been minimal. Furthermore, Lemma 4 immediately implies that $\langle w,s\rangle$ and $\langle u,t\rangle$ are also superbubbloids, i.e., neither $\langle w,u\rangle$ nor $\langle s,t\rangle$ is a superbubble in the situation of Lemma 5. Figure 2 shows a graph in which all (weak) superbubbloids and superbubbles are indicated.

### Reduction to auperbubble finding in DAGs

Theorem 1 guarantees that every weak superbubbloid and thus every superbubble in *G*(*V*, *E*) is completely contained within one of induced subgraphs *G*[*C*], $C\in \mathfrak {S}$. It does not guarantee, however, that a superbubble in *G*[*C*] is also a superbubble in *G*. This was already noted in [7]. This fact suggests to augment the induced subgraph *G*[*C*] of *G* by an artificial source *a* and an artificial sink *b*.

#### **Definition 5**

The augmented graph ${\tilde{G}}(C)$ is constructed from *G*[*C*] by adding the artificial source *a* and the artificial sink *b*. There is an edge (*a*, *x*) in ${\tilde{G}}(C)$ whenever $x\in C$ has an incoming edge from another component in *G* and there is an edge (*x*, *b*) whenever $x\in C$ has an outgoing edge to another component of *G*.

Since $G[{\bar{V}}]$ is acyclic, *a* has only outgoing edges and *b* only incoming ones, it follows that the augmented graph ${\tilde{G}}({\bar{V}})$ is also acyclic.

#### **Lemma 8**

$\langle s,t\rangle$ *is a weak superbubbloid in G if and only if it is a weak superbubbloid of* ${\tilde{G}}(C)$ *or a superbubbloid in* ${\tilde{G}}({\bar{V}})$ *that does not contain an axiliary source a or an auxiliary sink b.*

#### *Proof*

First assume that $\langle s,t\rangle$ is an induced subgraph of the strongly connected component *G*[*C*] of *G*. By construction, *G*[*C*] is also a strongly connected component of ${\tilde{G}}(C)$. Thus reachability within *C* is the same w.r.t. *G* and ${\tilde{G}}(C)$. Also by construction, a vertex $w\notin C$ is reachable from $x\in C$ in *G* if an only of *b* is reachable from *x* in ${\tilde{G}}(C)$. Similarly, a vertex $x\in C$ is reachable from $w\notin C$ if and only if *x* is reachable from *a*. Hence $\langle s,t\rangle$ is a (weak) superbubbloid w.r.t. *G* if and only if it a weak superbubbloid w.r.t. $\tilde{G}(C)$. For the special case that $\langle s,t\rangle$ is an induced subgraph of the acyclic graph $G[{\bar{V}}]$ we can argue in exactly the same manner.

For strongly connected components *C*, the graph ${\tilde{G}}(C)$ contains exactly 3 strongly connected components whose vertex sets are *C* and the singletons $\{a\}$ and $\{b\}$. Since (*a*, *b*) is not an edge in ${\tilde{G}}(C)$, every weak superbubbloid in ${\tilde{G}}(C)$ is contained in *G*[*C*] and hence contains neither *a* nor *b*. Superbubbloids containing *a* or *b* cannot be excluded for the acyclic component ${\tilde{G}}[{\bar{V}}]$, however. $\square$

It is possible, therefore, to find the weak superbubbloids of *G* by computing the weak superbubbloids not containing an artificial source or sink vertex in the augmented graphs. In the remainder of this section we show how this can be done efficiently.

The presentation below depends strongly on the properties of depth first search (DFS) trees and vertex orders associated with them. We thus briefly recall their relevant features. A *vertex order* is a bijection $\rho :V\rightarrow \{1,\dots ,|V|\}$. We write $\rho ^{-1}(i)$ is the vertex at the *i*-th position of the $\rho$-ordered vertex list. Later we will also need vertex sets that form intervals w.r.t. $\rho$. These will be denoted by $\rho ^{-1}([i,j]):=\{\rho ^{-1}(k)|i\le k\le j\}$ for a $\rho$-interval of vertices.

DFS on a strongly connected digraph *G* (exploring only along directed edges) is well known to enumerate all vertices starting from an arbitrary root [14]. The corresponding DFS tree consists entirely of edges of *G* pointing away from the root. In the following we will reserve the symbol $\rho$ for the reverse postorder of the DFS tree *T* in a strongly connected graph. Edges of *G* can be classified relative to a given DFS tree *T* with root *x*. By definition, all tree edges (*u*, *v*) are considered to be oriented away from the root *w*; hence $\rho (u)<\rho (v)$. An edge $(u,v)\in E(G)$ is a *forward edge* if *v* is reachable from *u* along a path consisting of tree edges, hence it satisfied $\rho (u)<\rho (v)$. The edge (*u*, *v*) is a *backward edge* if *u* is reachable from *v* along a path of consisting of tree edges, hence $\rho (u)>\rho (v)$. For remaining, so-called cross edges have no well-defined behavior w.r.t. $\rho$. We refer to [14, 15] for more details on depth first search, DFS trees, and the associated vertex orders.

A *topological sorting* of a directed graph order $\pi$ of *V* such that $\pi (u)<\pi (v)$ holds for every directed (*u*, *v*) [16]. Equivalently, $\pi$ is a topological sorting if there are no backward edges. A directed graph admits a topological sorting if and only if it is a DAG. In particular, if *v* is reachable from *u* then $\pi (u)<\pi (v)$ must hold. In a DAG, a topological sorting can be obtained as the reverse postorder of an arbitrary DFS tree that is constructed without considering the edge directions in *G* [15].

#### **Lemma 9**

*Let G be a strongly connected digraph,* $\langle s,t\rangle$ *be a weak superbubbloid in G,* $w\notin \langle s,t\rangle,$ *and* $\rho$ *the inverse postorder of a DFS tree* *T* *rooted at w. Then the induced subgraph* $\langle s,t\rangle$ *of G contains no backward edge w.r.t.* $\rho$ *except possibly (t, s).*

#### *Proof*

Let *T* be a DFS tree rooted in *T* and let $\delta$ denote the preordering of *T*. First we rule out $\delta (s)>\delta (t).$ Since *t* cannot be reached from anywhere along a path that does not contain *s*, this is only possible if $\rho (t)=1$, i.e., if *t* is the root of DFS tree *T*. This contradicts the assumption that $\rho (w)=1$ for some *w* outside $\langle s,t\rangle$. Hence $\delta (s)<\delta (t)$. The DFS tree *T* therefore contains a directed path from *s* to *t*. Since interior vertices of $\langle s,t\rangle$ are only reachable through *s* and reach outside only through *t*, it follows that the subtree $T^*$ of *T* induced by $\langle s,t\rangle$ is a tree and only *s* and *t* are incident to edges of *T* outside of $\langle s,t\rangle$. In the DFS reverse postorder $\rho$ we therefore have $\rho (s)<\rho (u)<\rho (t)$ for every vertex *u* interior to $\langle s,t\rangle$, and either $\rho (w)<\rho (s)$ or $\rho (w)>\rho (t)$ for all *w* outside of $\langle s,t\rangle$. The graph $G_{st}$ obtained from $\langle s,t\rangle$ by removing the possible (*t*, *s*) edge is a DAG, the subtree $T^*$ is a DFS tree on $G_{st}$, whose reverse postorder $\rho ^*$ is collinear with *rho*, i.e., $\rho ^*(u)<\rho ^*(v)$ holds whenever $\rho (u)<\rho (v)$. Therefore, there are no back-edges in $G_{st}$. $\square$

Lemma 9 is the key prerequisite for constructing an acyclic graph that contains all weak superbubbles of $\tilde{G}(C)$. Similar to the arguments above, however, we cannot simply ignore the backward edges. Instead, we will again add edges to the artificial source and sink vertices.

#### **Definition 6**

Given a DFS tree *T* with a root $w=\rho ^{-1}(1)$ that is neither an interior vertex nor the exit of a weak superbubbloid of ${\tilde{G}}(C)$, the auxiliary graph ${\hat{G}}(C)$ is obtained from ${\tilde{G}}(C)$ by replacing every backward edge (*v*, *u*) with respect to $\rho$ in ${\tilde{G}}(C)$ with both an edge (*a*, *u*) and an edge (*v*, *b*).

**Fig. 3:**
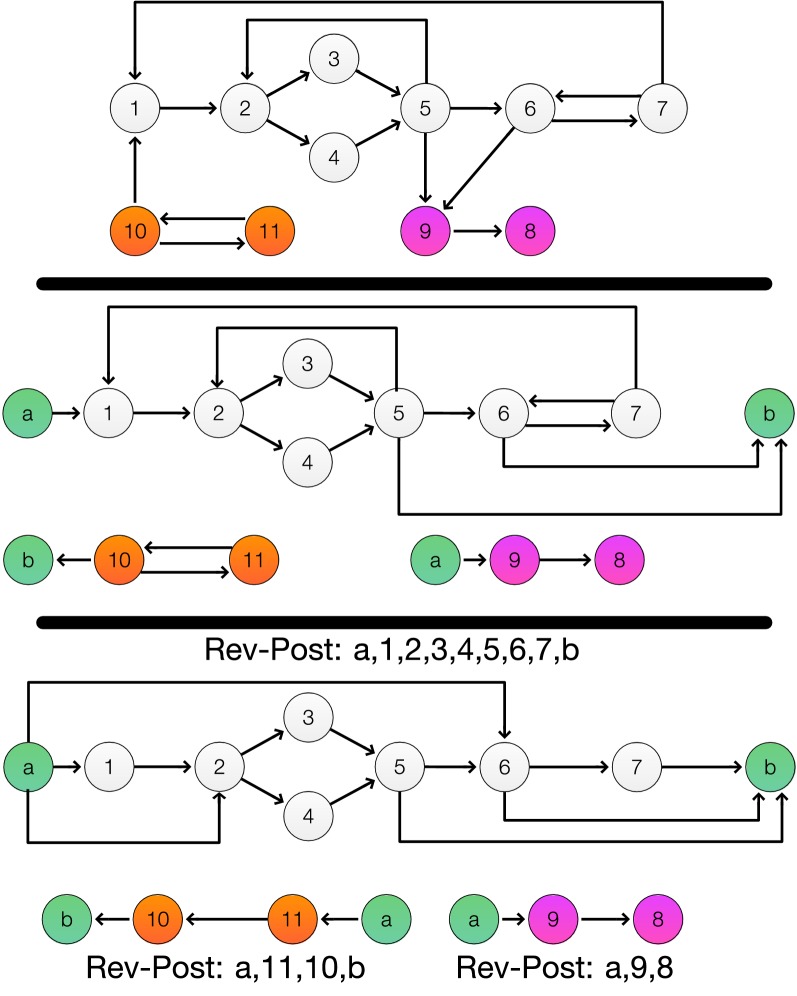
Example for the construction of ${\hat{G}}(C)$ from *G* (top). The graph *G* has two non-trivial SCCs (indicated by the white and orange vertices, resp.). In addition, there and two singleton SCCs (purple vertices) from which ${\tilde{G}}({\bar{V}})$ is constructed. The middle panel shows the graphs ${\tilde{G}}(C)$. Each is obtained by adding the artificial source and sink vertices *a* and *b*. The artificial source of the second SCC has no incident edge and in the DAG ${\tilde{G}}({\bar{V}})$ the artificial sink *b* has no incoming edge. These vertices are not shown since only the connected components containing *C* or ${\bar{V}}$ are of interest. The edges (10, 1), (5, 9) and (6, 9) in *G* form connections between the SCCs and the DAG, resp. Hence they are replaced by corresponding edges to an artificial source or artificial sink vertex according to Definition 5. The bottom panel shows the graphs ${\hat{G}}(C)$ obtained with the help of DFS searches. The reverse post ordering is shown. In the case of the second SCC, the artificial source *a* is connected to 11 as described in Corollary 6. The back edges (5, 2), (7, 1), (7, 6) and (10, 11) are then replaced with the corresponding edge to *a* and from *b* as prescribed by Definition 6. The tree graphs have the same superbubbles as *G*

Note that Definition 6 implies that all backward edges (*u*, *v*) of ${\tilde{G}}(C)$ are removed in ${\hat{G}}(C)$. As a consequence, ${\hat{G}}(C)$ is acyclic. The construction of ${\hat{G}}$ is illustrated in Fig. 3.

#### **Lemma 10**

*Let C be a strongly connected component of G and let T be a DFS tree on* ${\tilde{G}}(C)$ *with a root* $w=\rho ^{-1}(1)$ *that is neither an interior vertex nor the exit of a weak superbubbloid of G. Then* $\langle s,t\rangle$ *with* $s,t\in C$ *is a weak superbubble of G contained in* ${\tilde{G}}(C)$ *if and only if* $\langle s,t\rangle$ *is a superbubble in* ${\hat{G}}(C)$ *that does not contain the auxiliary source a or the auxiliary sink b.*

#### *Proof*

Assume that $\langle s,t\rangle$ is a weak superbubble in ${\tilde{G}}(C)$ that does not contain *a* or *b*. Lemma 8 ensures that this is equivalent to $\langle s,t\rangle$ being a weak superbubble of *G*. By Lemma 9, $\langle s,t\rangle$ contains no backward edges in ${\tilde{G}}(C)$, with the possible exception of the edge (*t*, *s*). Since ${\tilde{G}}(C)$ and ${\hat{G}}(C)$ by construction differ only in the backward edges, the only difference affecting $\langle s,t\rangle$ is the possible insertion of edges from *a* to *s* or from *t* to *b*. Neither affects a weak superbubble, however, and hence $\langle s,t\rangle$ is a superbubble in ${\hat{G}}(C)$.

Now assume that $\langle s,t\rangle$ is a superbubble in ${\hat{G}}(C)$ with vertex set $U_{st}$ and $a,b\notin U_{st}$. Since the restriction of ${\hat{G}}(C)$ to *C* is by construction a subgraph of ${\tilde{G}}(C)$, we know that reachability within *C* w.r.t. to ${\hat{G}}(C)$ implies reachability w.r.t. ${\tilde{G}}(C)$. Therefore $U_{st}$ satisfies (S.i) and (S.ii) also w.r.t. ${\tilde{G}}(C)$. Therefore, if $\langle s,t\rangle$ is not a weak superbubble in ${\tilde{G}}(C)$ then there must be a backwards edge (*x*, *v*) or a backward edge (*v*, *x*) with *v* in the interior of $\langle s,t\rangle$. The construction of ${\hat{G}}(C)$, however, ensures that ${\hat{G}}(C)$ then contains an edge (*a*, *v*) or (*v*, *b*), respectively, which would contradict (S.iii), (S.iv), or acyclicity (in case $x\in U_{st}$) and hence (S.v). Therefore $\langle s,t\rangle$ is a superbubble in ${\hat{G}}(C)$. $\square$

The remaining difficulty is to find a vertex *w* that can safely be used a root for the DFS tree *T*. In most cases, one can simply set $\rho (a)=1$ since Lemma 8 ensures that *a* is not part of a weak superbubbloid of *G*. However, there is no guarantee that an edge of the form (*a*, *w*) exists, in which case ${\tilde{G}}(C)$ is not connected. Thus another root for the DFS tree must be chosen. A closer inspection shows that three cases have to be distinguished:

A. *a* has an out-edge. In this case we can choose *a* as the root of the DFS tree, i.e., $\rho (a)=1$.
B. *a* has no edge, but there *b* has an in-edge. In this case we have to identify vertices that can only be entrances of a superbubble. These can then be connected with the artificial source vertex without destroying a superbubble.
C. Neither *a* nor *b* have edges. The case requires special treatment.

In order to handle case (B), we use the following

#### **Lemma 11**

*Let a and b be the artificial source and sink of* ${\tilde{G}}(C).$ *Let* $a'$ *and* $b'$ *be a successor of a and a predecessor of b, respectively. Then*

i) $a'$ *is neither an interior vertex nor the exit of a superbubble.*
ii) A predecessor $a''$* of *$a'$* is neither an interior vertex nor an entrance of a superbubble.*
iii) $b'$ *is neither an interior vertex nor the entrance of a superbubble.*
iv) *A successor* $b''$ *of* $b'$ *is neither an interior vertex nor an exit of a superbubble.*

#### *Proof*

If $a'$ is contained in a superbubble, it must be the entrance, since otherwise its predecessor, the artificial vertex *a* would belong to the same superbubble. If $a''$ is in the interior of an entrance, the $a'$ would be an interior vertex of a superbubble, which is impossible by (i). The statements for *b* follow analogously. $\square$

#### **Corollary 6**

*If b has an inedge in* ${\tilde{G}}(C),$ *then every successor* $b''\ne b$ *of every predecessor* $b'$ *of b can be used a root of the DFS search tree. At least one such vertex exists.*

#### *Proof*

By assumption, *b* has at least one predecessor $b'$. Since *G*[*C*] is strongly connected, $b'$ has at least one successor $b''\ne b$, which by Lemma 11(iv) is either not contained in a superbubble or is the entrance of a superbubble. $\square$

The approach sketched above fails in case (C) because there does not seem to be an efficient way to find a root for DFS tree that is guaranteed not to be an interior vertex or the exit of a (weak) superbubbloid. Sung et al. [7] proposed the construction of a more complex auxiliary DAG *H* that not only retains the superbubbles of *G*[*C*] but also introduces additional ones. Then all weak superbubbles in *H*(*G*) are identified and tested whether they also appeared in *G*[*C*].

#### **Definition 7**

(*Sung graphs*) Let *G* be a strongly connected graph with a DFS tree *T* with root *x*. The vertex set $V(H)=V'{{\dot{\cup }}} V''{\dot{\cup }} \{a,b\}$ consists of two copies $v'\in V'$ and $v''\in V''$ of each vertex $v\in V(G)$, a source *a*, and a sink *b*. The edge set of *H* comprises four classes of edges: (i) edges $(u',v')$ and $(u'',v'')$ whenever (*u*, *v*) is a forward edge in *G* w.r.t. *T*. (ii) edges $(u',v'')$ whenever (*u*, *v*) is a backward edge in *G*. (iii) edges $(a,v')$ whenever (*a*, *v*) is a edge in *G* and (iv) edges $(v'',b)$ whenever (*v*, *b*) is a edge in *G*.

The graph *H* is a connected DAG since a topological sorting on *H* is obtained by using the reverse postorder of *T* within each copy of *V*(*G*) and placing the first copy entirely before the second. We refer to [7] for further details.

The graph *H* contains two types of weak superbubbloids: those that contain no backward edges w.r.t. *T*, and those that contain backward edges. Members of the first class do not contain the root of *T* by Lemma 9 and hence are also superbubbles in *G*. Every weak superbubble of this type is present (and will be detected) in both $V'$ and $V''$. A weak superbubble with backward edge has a “front part” in $V'$ and a “back part” in $V''$ and appears exactly once in *H*. The vertex sets $V'$ and $V''$ are disjoint. It is possible that *H* contains superbubbles that have duplicated vertices, i.e., vertices $v'$ and $v''$ deriving from the same vertex in *V*. These candidates are removed together with one of the copies of superbubbles appearing in both $V'$ and $V''$. We refer to this filtering step as *Sung filtering* as it was proposed in [7].

This construction is correct in case (C) if there are no other edges connecting *G*[*C*] within *G*. The additional connections to *a* and *b* introduced to account for edges that connect *G*[*C*] to other vertices in *G*, may fail. To see this, consider an interior vertex $v'$ in a superbubble $\langle s,t\rangle$ with a backward edge. It is possible that its original has an external out edge and thus *b* should be connected to $v'$. This is not accounted for in the construction of *H*, which required that $V'$ is connected to *a* only, and $V''$ is connected to *b* only. These ”missing” edges may introduce false positive superbubbles as shown in Fig. 1.

This is not a dramatic problem because it is easy to identify the false positives: it suffices to check whether there is an edge (*x*, *w*) or (*w*, *y*) with $w\notin U_{st}$, $x\in U_{st}\backslash\{t\}$ and $y\in U_{st}\backslash\{s\}$. Clearly, this can be achieved in linear total time for all superbubble candidates $U_{st}$, providing a easy completion for the algorithm of Sung et al. [7]. Our alternative construction eliminates the need for this additional filtering step. 
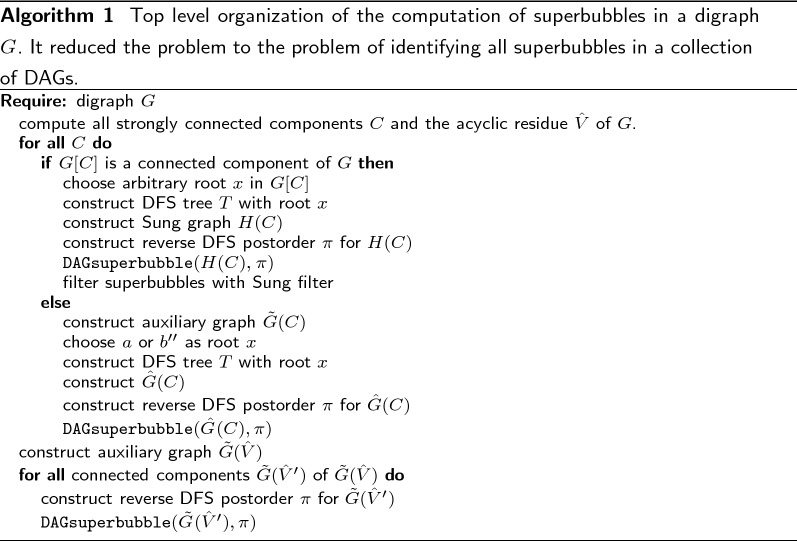


#### **Lemma 12**

*The (weak) superbubbles in a digraph G(V, E) can be identified in* $O(|V|+|E|)$ *time using Algorithm 1 provided the (weak) superbubbles in a DAG can be found in linear time.*

#### *Proof*

The correctness of Algorithm 1 is an immediate consequence of the discussion above. Let us briefly consider its running time. The strongly connected components of *G* can be computed in linear, i.e., $O(|V|+|E|)$ time [14, 17, 18]. The cycle-free part $G[{\hat{V}}]$ as well as its connected components [19] are also obtained in linear time. The construction of directed (to construct *T*) or undirected DFS search (to construct $\pi$ in a DAG) also require only linear time [14, 15], as does the classification of forward and backward edges. The construction of the auxiliary DAGs ${\hat{G}}(C)$ and *H*(*C*) and the determination of the root for the DFS searches is then also linear in time. Since the vertex sets considered in the auxiliary DAGs are disjoint in *G*, we conclude that the superbubbles can be identified in linear time in arbitrary digraph if the problem can be solved in linear time in a DAG. $\square$

The algorithm of Brankovic et al. [8] shows that this is indeed the case.

#### **Corollary 7**

*The (weak) superbubbles in a digraph G(V, E) can be identified in* $O(|V|+|E|)$ *time using Algorithm 1.*

**Fig. 4:**
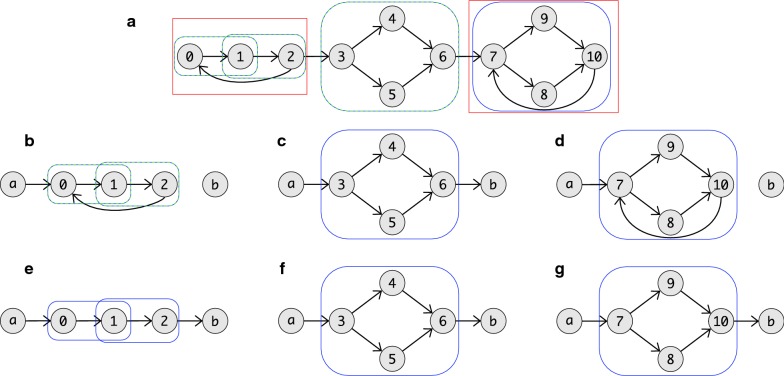
An example graph that is transformed in three DAGs after Algorithm 1. In every graph are the weak superbubbles (blue) and all superbubbles (green) marked. In **a** is the original graph shown. Here are the non singleton SCC are marked with a red square. In **b**, **d** are ${\tilde{G}}(C)$ for the SCC are shown and in **c** is ${\tilde{G}}({\hat{V}})$ shown. In **e** and **g** are ${\hat{G}}(C)$ are shown and in **f** again ${\tilde{G}}({\hat{V}})$ because no ${\hat{G}}({\hat{V}})$ is needed. In the three DAGs are no differentiation between superbubbles and weak superbubbles are possible because they are equivalent in DAGs. So here are only the weak superbubbles are marked. Note that in **g** the weak superbubble $\langle 7,10\rangle$ of **D** is now also a superbubble. However, this can be simple detected later by checking if an edge (10, 7) exists

In the following section we give a somewhat different account of a linear time algorithm for superbubble finding that may be more straightforward than the approach in [8], which heavily relies on range queries. An example graph as the different auxiliary graphs are shown in Fig. 4.

### Detecting superbubbles in a DAG

The identification of (weak) superbubbles is drastically simplified in DAGs since acyclicity, i.e., (S3), and thus (S.v), can be taken for granted. In particular, therefore, every weak superbubbloid is a superbubbloid. A key result of [8] is the fact that there are vertex orders for DAGs in which all superbubbles appear as intervals. The proof of Proposition 2 does not make use the minimality condition hence we can state the result here more generally for superbubbloids and arbitrary DFS trees on *G*:

#### **Proposition 2**

([8]) *Let G(V, E) be a DAG and let *$\pi$ *be the reverse postorder of a DFS tree of G. Suppose *$\langle s,t\rangle$ *is a superbubbloid in G. Then*

i) *Every interior vertex u of* $\langle s,t\rangle$ *satisfied* $\pi (s)<\pi (u)<\pi (t).$
ii) *If* $w\not \in \langle s,t\rangle$ *then either* $\pi (w)<\pi (s)$ *or* $\pi (t)<\pi (w).$

The following two functions were also introduced in [8]:

1 $$\begin{aligned} \begin{aligned} \mathop {\mathbf {OutParent}}(v)&:= {\left\{ \begin{array}{ll} -1&{} \text {if no } (u, v) \in E \text { exists} \\ \min (\{\pi (u) | (u, v) \in E\})&{}\text {otherwise} \end{array}\right. }\\ \mathop {\mathbf {OutChild}}(v)&:= {\left\{ \begin{array}{ll} \infty &{} \text {if no } (v, u) \in E \text { exists} \\ \max (\{\pi (u) | (v, u) \in E\})&{}\text {otherwise} \end{array}\right. } \end{aligned} \end{aligned}$$

We slightly modify the definition here to assign values also to the sink and source vertices of the DAG *G*. The functions return the predecessor and successor of *v* that is furthest away from *v* in terms of the DFS order $\pi$. It is convenient to extend this definition to intervals by setting

2 $$\begin{aligned} \begin{aligned} \mathop {\mathbf {OutParent}}([i,j])&:= \min \{\mathop {\mathbf {OutParent}}(v)\mid v \in \pi ^{-1}([i,j])\} \\ \mathop {\mathbf {OutChild}}([i,j])&:= \max \{\mathop {\mathbf {OutChild}}(v) \mid v \in \pi ^{-1}([i,j])\} \end{aligned} \end{aligned}$$

A main result of this contribution is that superbubbles are characterized completely by these two functions, resulting in an alternative linear-time algorithm for recognizing superbubbles in DAGs that also admits a simple proof of correctness. To this end we will need a few simple properties of the $\mathop {\mathbf {OutParent}}$ and $\mathop {\mathbf {OutChild}}$ functions for intervals. First we observe that $[k,l]\subseteq [i,j]$ implies the inequalities

3 $$\begin{aligned} \begin{aligned} \mathop {\mathbf {OutParent}}([k,l])&\ge \mathop {\mathbf {OutParent}}([i,j]) \\ \mathop {\mathbf {OutChild}}([k,l])&\le \mathop {\mathbf {OutChild}}([i,j]) \end{aligned} \end{aligned}$$

A key observation for our purposes is the following

#### **Lemma 13**

*If* $$\mathop {\mathbf {OutChild}}([i,j-1])\le j<\infty$$ *then*

i) $\pi ^{-1}(j)$ *is the only successor of* $\pi ^{-1}(j-1);$
ii) $\pi ^{-1}(j)$ *is reachable from every vertex* $v\in \pi ^{-1}([i,j-1]);$
iii) e*very path from some* $v\in \pi ^{-1}([i,j-1])$* to a vertex* $w\notin \pi ^{-1}([i,j-1])$* contains* $\pi ^{-1}(j).$

#### *Proof*

(i) By definition $\pi ^{-1}(j-1)$ has at least one successor. On the other hand, all successor of $\pi ^{-1}$ after $j-1$ are by definition not later than *j*. Hence $\pi ^{-1}(j)$ is uniquely defined.
(ii) We proceed by induction w.r.t. the length of the interval $[i,j-1]$. If $i=j-1$, i.e., a single vertex, the assertion (ii) is obviously true. Now assume that the assertion is true for $[i+1,j]$. By definition of $\mathop {\mathbf {OutChild}}$, *i* has a successor in $[i+1,j]$, from which $\pi ^{-1}(j)$ is reachable.
(iii) Again, we proceed by induction. The assertion holds trivially for single vertices. Assume that the assertion is true for $[i+1,j]$. By definition of $\mathop {\mathbf {OutChild}}$, every successor *u* of $\pi ^{-1}(i)$ is contained in $\pi ^{-1}([i+1,j])$. By induction hypothesis, every path from *u* to a vertex $w\notin \pi ^{-1}([i-1,j-1])$ contains $\pi ^{-1}(j)$, and also all path from $\pi ^{-1}(i)$ to $w\notin \pi ^{-1}([i,j-1])$ run through $\pi ^{-1}(j)$.

$$\square$$

It is important to notice that Lemma 13 depends crucially on the fact that $\pi$, by construction, is a reverse postorder of a DFS tree. It does not generalize to arbitrary topological sortings.

Replacing successor by predecessor in the proof of Lemma 13 we obtain

#### **Lemma 14**

*If* $$\mathop {\mathbf {OutParent}}([i+1,j])\ge i>-1$$ *then*

i) $\pi ^{-1}(i)$ *is the only predecessor of* $\pi ^{-1}(i+1);$
ii) *Every vertex* $v\in \pi ^{-1}([i+1,j])$ *is reachable from* $\pi ^{-1}(i);$
iii) *Every path from* $w\notin \pi ^{-1}([i+1,j])$ *to a vertex* $v\in \pi ^{-1}([i+1,j])$ *contains* $\pi ^{-1}(i).$

Let us now return to the superbubbloids. We first need two simple properties of the $\mathop {\mathbf {OutParent}}$ and $\mathop {\mathbf {OutChild}}$ function for individual vertices:

#### **Lemma 15**

*Let* $\langle s,t\rangle$ *is a superbubbloid in a DAG G. Then*

i) *v is an interior vertex of* $\langle s,t\rangle$* implies* $\pi (s)\le \mathop {\mathbf {OutParent}}(v)$* and *$\mathop {\mathbf {OutChild}}(v)\le \pi (t)$.
ii) $$\pi (s)\le \mathop {\mathbf {OutParent}}(t)$$* and* $$\mathop {\mathbf {OutChild}}(s)\le \pi (t)$$.
iii) *If* $w\notin \langle s,t\rangle$ *then* $\mathop {\mathbf {OutParent}}(w)< \pi (s)$ *or* $\mathop {\mathbf {OutParent}}(w)\ge \pi (t),$ *and* $\mathop {\mathbf {OutChild}}(w)\le \pi (s)$ *or* $\mathop {\mathbf {OutChild}}(w)>\pi (t).$

#### *Proof*

(i) The matching property (S2) implies that for every successor *x* and predecessor *y* of an interior vertex *v* there is a path within the superbubble from *s* to *x* and from *y* to *t*, respectively. The statement now follows directly from the definition.
(ii) The argument of (i) applies to the successors of *s* and the predecessors of *t*.
(iii) Assume, for contradiction, that $\pi (s)\le \mathop {\mathbf {OutParent}}(w)<\pi (t)$ or $\pi (s)<\mathop {\mathbf {OutChild}}(w)\le \pi (t)$. Then Proposition 2 implies that *w* has a predecessor $v'$ or successor $v''$ in the interior of the superbubble. But then $v'$ has a successor (namely *w*) outside the superbubble, or $v''$ has a predecessor (namely *w*) inside the superbubble. This contradicts the matching condition (S2).

$$\square$$

#### **Theorem 2**

*Let G be a DAG and let* $\pi$ *be the reverse postorder of a DFS tree on G. Then* $\langle s,t\rangle$ *is a superbubbloid if and only if the following conditions are satisfied:*

1. $$\mathop {\mathbf {OutParent}}([\pi (s)+1,\pi (t)])=\pi (s)$$ *(predecessor property)*
2. $$\mathop {\mathbf {OutChild}}([\pi (s),\pi (t)-1])=\pi (t)$$ *(successor property)*

#### *Proof*

Suppose $\mathop {\mathbf {OutParent}}$ and $\mathop {\mathbf {OutChild}}$ satisfy (F1) and (F2). By (F1) and Lemma 13(ii) we known that *t* is reachable from every vertex in *v* with $\pi (s)\le \pi (v)<\pi (t)$. Thus the reachability condition (S1) is satisfied. Lemma 13(iii) implies that any vertex *w* with $\pi (w)<\pi (s)$ or $\pi (w)>\pi (t)$ is reachable from *v* only through a path that runs through *t*. The topological sorting then implies that *w* with $\pi (w)<\pi (s)$ is not reachable from at all since *w* is not reachable from *t*. Hence $U_{st}= \pi ^{-1}([\pi (s),\pi (t)]$. By (F2) and Lemma 14(ii) every vertex *v* with $\pi (s)<\pi (v)\le \pi (t)$, i.e., is reachable from *s*. Lemma 14(ii) implies that *v* is reachable from a vertex *w* with $\pi (w)<\pi (s)$ or $\pi (w)>\pi (t)$ only through paths that contain *s*. The latter are not reachable at all since *s* is not reachable from *w* with $\pi (w)>\pi (t)$ in a DAG. Thus $U^+_{ts}=\pi ^{-1}([\pi (s),\pi (t)]=U_{st}$, i.e., the matching condition (S2) is satisfied.

Now suppose (S1) and (S2) holds. Lemma 15 implies that $\mathop {\mathbf {OutParent}}([\pi (s)+1,\pi (t)])\ge \pi (s)$. Since some vertex $v'\in \langle s,t\rangle$ must have *s* as its predecessor we have $\mathop {\mathbf {OutParent}}([\pi (s)+1,\pi (t)])=\pi (s)$, i.e., (F1) holds. Analogously, Lemma 15 implies $\mathop {\mathbf {OutChild}}([\pi (s),\pi (t)-1])\le \pi (t)$. Since there must be some $v'\in \langle s,t\rangle$ that has *t* as its successor, we must have $\mathop {\mathbf {OutChild}}([\pi (s),\pi (t)-1])=\pi (t)$, i.e. (F2) holds. $\square$

We now proceed to showing that the possible superbubbloids and superbubbles can be found efficiently, i.e., in linear time using only the reserve postorder of the DFS tree and the corresponding functions $\mathop {\mathbf {OutChild}}$ and $\mathop {\mathbf {OutParent}}$. As an immediate consequence of (F2) and Lemma 13, we have the following necessary condition for exits:

#### **Corollary 8**

*The exit t of superbubbloid* $\langle s,t\rangle$ *satisfies* $\mathop {\mathbf {OutChild}}(\pi ^{-1}(\pi (t)-1))=\pi (t).$

We now use the minimality condition of Definition 2 to identify the superbubbles among the superbubbloids.

#### **Lemma 16**

*If t is the exit of a superbubbloid, then there is also the exit of a superbubble* $\langle s,t\rangle$ *whose entrance s is vertex with the largest value of* $\pi (s)<\pi (t)$ *such that (F1) and (F2) is satisfied.*

#### *Proof*

Let $\langle s,t\rangle$ be a superbubbloid. According to Definition 2, $\langle s,t\rangle$ is a superbubble if there is no superbubbloid $\langle s',t\rangle$ with $\pi (s)<\pi (s')<\pi (t)$, i.e., there is no vertex $s'$ with $\pi (s')>\pi (s)$ such that (F1) and (F2) is satisfied. $\square$

#### **Lemma 17**

*Suppose* $\pi (s)\le \pi (v)<\pi (t)$ *and* $\mathop {\mathbf {OutChild}}(v)>\pi (t).$ *Then there is no superbubbloid with entrance s and exit t.*

#### *Proof*

Suppose $\langle s,t\rangle$ is a superbubbloid. By construction, $\mathop {\mathbf {OutChild}}([\pi (s),\pi (t)-1])\ge \mathop {\mathbf {OutChild}}(v)>\pi (t)$, contradicting (F2). $\square$

#### **Corollary 9**

*If* $\langle s,t\rangle$ *is a superbubble, then there is no superbubbloid* $\langle s',t'\rangle$ *with exit* $t'\in \pi ^{-1}([\pi (s)+1,\pi (t)-1])$ *and entrance* $s'$ *with* $\pi (s')<\pi (s).$

#### *Proof*

This is an immediate consequence of Lemma 5, which shows that the intersection $\langle s,t\rangle \cap \langle s',t'\rangle$ would be a superbubbloid, contradicting minimality of $\langle s,t\rangle$. $\square$

#### **Corollary 10**

*If* $\langle s,t\rangle$ *and* $\langle s',t'\rangle$ *are two superbubbles with* $\pi (t')<\pi (t)$ *then either* $\pi (s')<\pi (t')<\pi (s)<\pi (t),$ *or* $\pi (s)<\pi (s')<\pi (t')<\pi (t).$

Thus superbubbles are either nested or placed next to each other, as already noted in [6]. Finally, we show that it is not too difficult to identify false exit candidates, i.e., vertices that satisfy the condition of Corollary 8 but have no matching entrance *s*.

#### **Lemma 18**

*Let* $\langle s,t\rangle$ *be a superbubble and suppose* $t'$ *is an interior vertex of* $\langle s,t\rangle.$ *Then there is a vertex v with* $\pi (s)\le \pi (v)<\pi (t')$ *such that* $\mathop {\mathbf {OutChild}}(v)>\pi (t').$

#### *Proof*

Suppose, for contradiction, that no such vertex *v* exists. Since $\langle s,t\rangle$ is superbubble by assumption, it follows that $\mathop {\mathbf {OutParent}}([\pi (s)+1,\pi (t')])=\pi (s)$ is correct and so (F1) satisfied for $\langle s, t'\rangle$. After no such *v* exists also $\mathop {\mathbf {OutChild}}([\pi (s),\pi (t')-1])\le \pi (t)$ is correct and so (F2) is satisfied. Thus $\langle s,t'\rangle$ is superbubbloid. By Lemma 4 $\langle t',t\rangle$ is also a superbubbloid, contradicting the assumption. $\square$

Taken together, these observations suggest to organize the search by scanning the vertex set for candidate exit vertices *t* in reverse order. For every such *t*, one would then search for the corresponding entrance *s* such that the pair *s*, *t* fulfills (F1) and (F2). Using eq.(3) one can test (F2) independently for each *v* by checking whether $\mathop {\mathbf {OutChild}}(v)\le \pi (t)$. Checking for (F1) requires that the interval $[\pi (s)+1,\pi (t)]$ is considered. The value of its $\mathop {\mathbf {OutParent}}$ function can be obtained incrementally as the minimum of $\mathop {\mathbf {OutParent}}(v)$ and the $\mathop {\mathbf {OutParent}}$ interval of the previous step:

4 $$\begin{aligned} { \mathop {\mathbf {OutParent}}([\pi (v),\pi (t)]) = \min \left( \mathop {\mathbf {OutParent}}(v),\mathop {\mathbf {OutParent}}([\pi (v)+1,\pi (t)])\right) } \end{aligned}$$

By Lemma 16, the nearest entrance *s* to the exit *t* completes the superbubble. The tricky part is to identify all superbubbles in a single scan. Lemma 17 ensures that no valid entrance can be found for exit $t'$ if a vertex *v* with $\mathop {\mathbf {OutChild}}(v)>\pi (t')$ is encountered. In this case $t'$ can be discarded. Lemma 18 ensures that a false exit candidate $t'$ within a superbubble $\langle s,t\rangle$ candidate cannot “mask” the entrance *s* belonging to *t*, i.e., there is necessarily a vertex *v* satisfying $\mathop {\mathbf {OutChild}}(v)>\pi (t')$ with $\pi (s)<\pi (v)$.

It is natural therefore to use a stack $\mathbb {S}$ to hold the exit candidates. Since the $\mathop {\mathbf {OutParent}}$ interval explicitly refers to an exit candidate *t*, it must be re-initialized whenever a superbubble is completed or the candidate exit is rejected. More precisely, the $\mathop {\mathbf {OutParent}}$ interval of the previous exit candidate *t* must be updated. This is achieved by computing

5 $$\begin{aligned} { \mathop {\mathbf {OutParent}}[\pi (v),\pi (t)]= \min \left( \mathop {\mathbf {OutParent}}[\pi (v),\pi (t')],\mathop {\mathbf {OutParent}}[\pi (t')+1,\pi (t)]\right) } \end{aligned}$$

To this end, the value $\mathop {\mathbf {OutParent}}[\pi (t')+1,\pi (t)]$ is associated with *t* when $t'$ is pushed onto the stack. The values of $\mathop {\mathbf {OutParent}}$ intervals are not required for arbitrary intervals. Instead, we only need $\mathop {\mathbf {OutParent}}([\pi (t')+1,\pi (t)])$ with consecutive exit candidates $t'$ and *t*. Hence a single integer associated with each candidate *t* suffices. This integer initialized with $\mathop {\mathbf {OutParent}}(t)$ and is then advanced as described above to $\mathop {\mathbf {OutParent}}([\pi (v),\pi (t)])$. 
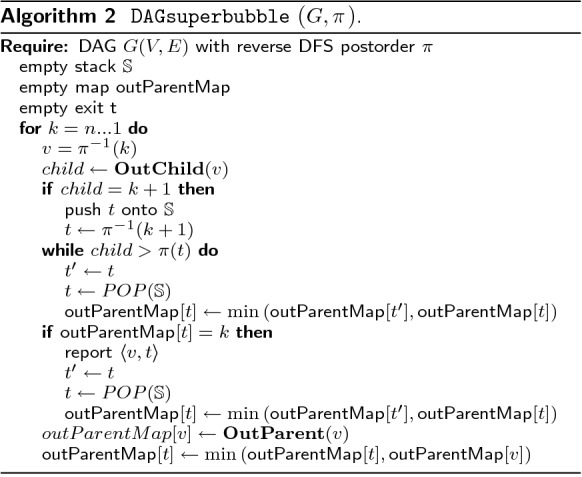


Algorithm 2 presents this idea in a more formal way.

#### **Lemma 19**

*Algorithm 2 identifies the superbubbles in a DAG G.*

#### *Proof*

Every reported candidate satisfied (F1) since $\mathop {\mathbf {OutParent}}([\pi (s)+1,\pi (t)])=\pi (s)$ is used to identify the entrance for the current *t*. Since $v\in \pi ^{-1}[\pi (s),\pi (t)-1]$ is checked for every $\mathop {\mathbf {OutChild}}(v)\le \pi (t)$, (F2) holds due to equ.(3) since by Lemma 13 this is equal to test the interval. Hence every reported candidate is a superbubbloid. By Lemma 16 $\langle s,t\rangle$ is minimal and thus a superbubble. Lemma 18 ensures that the corresponding entrance is identified for every valid exit *t*, i.e., that all false candidate exits are rejected before the next valid entrance in encountered. $\square$

#### **Lemma 20**

*The Algorithm 2 has time complexity* $\mathcal {O}(|V|+|E|).$

#### *Proof*

Given the reverse DFS postorder $\pi$, the for loop processes every vertex exactly once. All computations except $\mathop {\mathbf {OutChild}}(v)$, $\mathop {\mathbf {OutParent}}(v)$, and the while loop take constant time. This includes explicit the calculation of the minimum of two integer values that are needed to update of the intervals. The values of $\mathop {\mathbf {OutChild}}(v)$ and $\mathop {\mathbf {OutParent}}(v)$ are obtained by iterating over the outgoing or incoming edges of *v*, respectively, hence the total effort is $\mathcal {O}(|V|+|E|)$. Every iteration of the while loop removes one vertex from the stack $\mathbb {S}$. Since each vertex is pushed only $\mathbb {S}$ at most once, the total effort for the while loop is *O*(|*V*|). The total running time therefore is $\mathcal {O}(|V|+|E|)$. $\square$

Recalling the reverse DFS postorder $\pi$ can also be obtained in $\mathcal {O}(|V|+|E|)$ we have

#### **Corollary 11**

([8])* The superbubbles in a DAG can be identified in a linear time.*

**Fig. 5:**
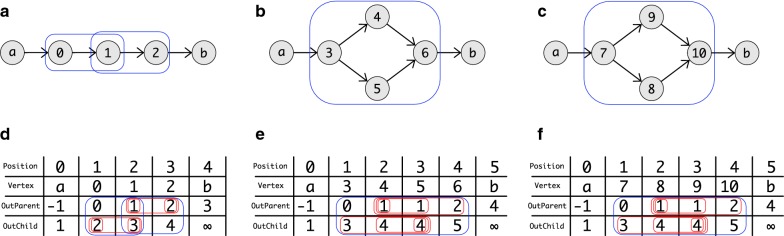
Some example DAGS and the The corresponding ordering and values for $\mathop {\mathbf {OutParent}}$ and $\mathop {\mathbf {OutChild}}$ are shown. The ordering starts for all graphs in *a*. In **a**–**c** the DAGs are shown. Here are the superbubbles are marked with a blue. In **d**−**f** are the ordering and values of $\mathop {\mathbf {OutParent}}$ and $\mathop {\mathbf {OutChild}}$ are shown. All intervals that fulfill (F1) or (F2) are marked red. The intervals that fulfill both and also the minimality criterion are marked blue. Note that by definition *a* and *b* can not be part of any superbubble and so they can not fulfill (F1) or (F2) so intervals that would contain *a* or *b* are not marked

Some example DAGs together with the values of $\mathop {\mathbf {OutChild}}$ and $\mathop {\mathbf {OutParent}}$ are shown in Fig. 5.

### Implementation

Algorithms 1 and 2 were implemented in Python and are available as *Linear Superbubble Detector*, LSD for short. LSD can be installed with **pip**.^1^ The source is available on GitHub.^2^ It is intended as a reference implementation emphasizing easy understanding rather than as a performance-optimized production tool. The underlying graph structures make use of NetworkX [20], which has the benefit that many input formats can be parsed easily.

To our knowlege, SUPBUB^3^ [8] is the only other publicly available implementation of a superbubble detector. Unfortunately, it has some bugs e.g., in the handling of successors in the DFS tree that leads to problems with superbubble with a backward edge. An analysis of the code shows, furthermore, that the construction of the auxiliary graphs strictly follows [7]. Hence it cannot serve as a reference implementation.

In order to compare our approach to the state of the art algorithm we re-implemented the workflow on Sung et al. [7] and Brankovic et al. [8] using the same python libraries. This allows a direct comparison that focusses on the algorithms rather than the differences between programming languages and compilers. The workflow can be subdivided into two separate tasks: (1) the construction of the DAGs, and (2) the recognition of superbubbles within the DAG. For the first task, we compare our approach and the algorithm of Sung et al. [7] augmented by a simple linear-time filter to detect the false positives. For the second part, we compare our stack-based approach with the range-query method of Brankovic et al. [8].

Table 1 summarized the empirical results for test data of different sizes taken from our recent work on supergenome coordinatization and the Stanford Large Network Dataset Collection [21]. Although the running times are comparable, we find that LSD consistently performs better than the alternative for both tasks. The combined improvement of LSD is a least a factor of 2 in the examples tested here. All results and methods are available in the git repository.^4^

**Table 1:** Comparison of running times

Data	*N*	*M*	*S*	Running times [s]			
				LSD	S + LSD+ f	LSD + B	S + B + f
Yeast	49,795	130,993	325	3	4	6	9
EU mail	265,214	420,045	13285	15	16	31	34
Slashdot	82,168	948,464	0	17	27	22	37
Amazon	403,394	3,387,388	3	60	86	87	158
Google	875,713	5,105,039	6477	94	127	144	254
Wikipedia	2,394,385	5,021,410	4737	147	171	385	418

The for combinations of algorithms compared here are: LSD (using the auxiliary graphs ${\hat{G}}{C}$ and the stack-based superbubble detector), S+LSD using Sung graphs with our stack-based detector plus a post-filter for the false positives, LSD+B using our graph construction with the range-query-based detector of [8], and S+B using the re-implementation of the state of the art method with the post-filter. All computations were performed on a 2.5GHz quad-core Intel Core i7 processor (Turbo Boost up to 3.7GHz) with 6MB shared L3 cache and 16GB of 1600MHz DDR3L onboard memory. Test data sets are taken from [4] and from the Stanford Large Network Dataset Collection [21]. The table lists their number *N* of vertices, *M* of edges and *S* of superbubbles

## Conclusion

We have re-investigated the mathematical properties of superbubbles and their obvious generalization, the weak superbubbloids. We not only re-derive foundational results, in particular Propositions 1 and 2 in a more concise way, we also identified a problems with auxiliary graphs proposed in [7] that lead to false positive superbubbles. Although these are not a fatal problem and can be recognized in a post-processing step without affecting the overall time-complexity, we have shown here that the issue can be avoided by using a different, in fact simpler, auxiliary graph that is already acyclic. Capitalizing on the fact that the superbubbles in a DAG can be listed in linear time [8], we show that problem of listing all superbubbles in an arbitrary digraph can indeed be solved in linear time. For the DAG case we proposed a conceptually simpler replacement for the algorithm of [8] that also has linear running time. With LSD we provide a reference implementation in python.

The mathematical analysis of superbubbles suggests to consider generalizations that allow possibly restricted sets of cycles within the “bubble” but retain the idea of an induced subgraph that cannot be transversed without passing through the entrance the exit. For instance, one might relax (S.v) an require only that an interior vertex *v* cannot be reached from *t* without passing through *s* and cannot reach *s* without passing through *t*. The false positives generated by the approach of Sung et al. [7] may also be considered a the prototype of a broader class of superbubble-like structures. It does not seem obvious, however, to characterize them beyond being induced acyclic subgraphs with a single source and a single sink vertex. An related structure that also generalizes superbubbles are maximal connected convex acyclic induced subgraphs [22]. Here, the vertex *U* set has the property that no two vertices $x,y\in U$ are connected by path that is not entirely contained in *U*.

## Footnotes

1 https://pypi.org/project/LSD-Bubble/.

2 https://github.com/Fabianexe/Superbubble.

3 https://github.com/Ritu-Kundu/Superbubbles.

4 https://github.com/Fabianexe/Superbubble.
